# A framework for conducting clinical trials involving 3D printing of medicines at the point-of-care

**DOI:** 10.1007/s13346-025-01868-y

**Published:** 2025-05-09

**Authors:** Carlos Javier Parramon-Teixido, Lucía Rodríguez-Pombo, Abdul W. Basit, Anna Worsley, Carme Cañete-Ramírez, Carmen Alvarez-Lorenzo, Maria Josep Cabañas-Poy, Alvaro Goyanes

**Affiliations:** 1 Pharmacy Department, Vall d’Hebron Hospital Universitari, Vall d’Hebron Barcelona Hospital Campus, Barcelona, Spain; 2 Departamento de Farmacología, Farmacia y Tecnología Farmacéutica, I+D Farma (GI-1645), Facultad de Farmacia, Instituto de Materiales (iMATUS) and Health Research Institute of Santiago de Compostela (IDIS), Universidade de Santiago de Compostela, Santiago de Compostela, 15782 Spain; 3 Department of Pharmaceutics, UCL School of Pharmacy, University College London, 29-39 Brunswick Square, London, WC1N 1AX UK; 4 FABRX Ltd, Henwood House, Henwood, Ashford, Kent, TN24 8DH UK; 5 FABRX Artificial Inteligente, Calle Enrique Vidal Abascal 7 Bajo, Santiago de Compostela, CP 15702 Spain

**Keywords:** 3D printing, Regulatory, Personalized medications, Paediatrics, Printed pharmaceuticals and formulations, Quality control

## Abstract

The integration of 3D printing (3DP) technologies into personalized medicine manufacture at the point-of-care is garnering significant interest due to its potential to create tailored drug products with precise dosages and other unique attributes. Both preclinical and clinical studies have demonstrated promising outcomes, including pharmacokinetic bioequivalence, improved patient acceptability, enhanced adherence, and the ability to produce consistent, reproducible dosage forms with accurate drug distribution. Some compounding pharmacies around the world are already incorporating 3DP into standard practice for simpler therapeutic treatments. However, further clinical evaluation is required for more complex treatments, such as multi-drug polypills. Conducting clinical trials involving 3DP technologies presents several challenges, including navigating evolving regulatory frameworks, addressing ethical and legal concerns, and complying with new point-of-care manufacturing guidelines. Although regulatory agencies are beginning to adapt their policies to accommodate 3DP, the absence of a comprehensive framework still creates uncertainty for pharmacists and healthcare providers. This article explores the planning and execution of clinical trials involving 3D printed medicines, with a focus on regulatory barriers, patient recruitment, compliance, and the integration of specialized equipment and expertise. It also discusses the implementation of 3DP for personalized drug manufacturing within hospital settings and offers guidance for obtaining clinical trial approval from the Spanish Agency for Medicine and Health Products (AEMPS). By providing these insights and recommendations, this article aims to support international harmonization and facilitate the adoption of 3DP technologies in clinical trials globally.

## Introduction

The availability of appropriately designed medications for all types of patient populations remains a notable challenge within the pharmaceutical landscape. Historically, the focus of pharmaceutical research, regulation, and formulation development has primarily been on adult male patients, often overlooking specific needs across different demographic groups [1, 2]. Many medicines on the market are not optimally designed for certain populations, such as paediatrics or elderly, leading to a limited range of suitable drug options that do not always meet specific requirements in terms of dosing and acceptability [1–4]. Doses are typically calculated based on factors such as an individual’s weight, body surface area, or specific pathological characteristics. In general, the physiological attributes of patients can vary significantly, emphasizing the need for tailored therapeutic approaches. It is well-known that different demographic groups may present unique needs or pathologies, which can significantly impact pharmacokinetics. Therefore, precise formulations and dosing are essential to ensure efficient and safe therapy for all patients. Reports such as the World Health Organization’s (WHO) “Make Medicines Child Size” highlight the broader necessity of improving access to medications that effectively address the health problems of underserved populations, like children [5]. Likewise, regulatory advancements, such as the European Union’s Paediatric Regulation (No 1901/2006) introduced in 2007, demonstrate ongoing efforts to enhance health outcomes across diverse groups [6].

While acceptability is essential for ensuring high adherence to prescribed treatments across all patient populations, it is especially important in paediatric patients [7]. Understanding patient adherence often involves an interplay of many factors that influence whether a patient successfully follows recommendations or completes a therapeutic program. Indeed, the “*Guideline on Pharmaceutical Development of Medicines for Paediatric Use*” released by the European Medicines Agency (EMA) provides formulation criteria and aspects to take into account [8]. Notably, there are significant differences compared to formulation development for adults, and challenges that must be considered are described; mainly, heterogeneity, precise and appropriate dosing, swallowing difficulties, palatability and acceptability, and excipient safety.

To overcome these problems related to the lack of medications, the use of unlicensed and off-label medicines (i.e., those prescribed and/or administered outside the terms of their marketing authorisation) is frequent in children due to their exclusion from trials during the drug development process [9]. Medicines are usually manually compounded within hospital or community pharmacies with unique attributes tailored to each patient, such as specific dosage or administration form. Compounded medicines are intended for an individual patient and they are prepared by a pharmacist through the combination and customization of active pharmaceutical ingredients (APIs) and excipients [10]. Compounding remains a vital practice for pharmacist and a fundamental pillar to guarantee personalized treatment according to patient’s requirements [11, 12].

Notwithstanding, pharmaceutical compounding exhibits some limitations [13, 14]. It is time-consuming, resource-intensive, not exempt to dosing errors, and sometimes an inflexible approach to meet continuous dose changes. In addition, compounded formulations may present adherence challenges when the final dosage form is not well accepted by the patient; e.g. capsules may be unsuitable for individuals with swallowing difficulties. Liquid formulations are commonly regarded as the preferred oral dosage form for compounding in paediatric patients. However, these formulations often carry a bitter or an unpleasant-tasting due to the drug, and their color and even their packaging make them unappealing and may generate resistance to take it, potentially negatively impact treatment adherence [15, 16]. While these liquid formulations offer flexibility in dosing, achieving precise and consistent dosing can be challenging due to issues such as inhomogeneity and poor palatability. Patients may spit out the medication or sometimes masking its taste with food, resulting in an increased risk of inaccurate dosing due to incomplete uptake, bioavailability changes or even aversion to these foods. These challenges in adherence and dosing accuracy have been associated with hospital admissions and higher healthcare cost [16–18], underscoring the critical need for addressing these issues in compounding. Considering these problems, it is essential to explore alternative formulations or methods that can overcome the main limitations of conventional pharmaceutical compounding.

**Fig. 1:**
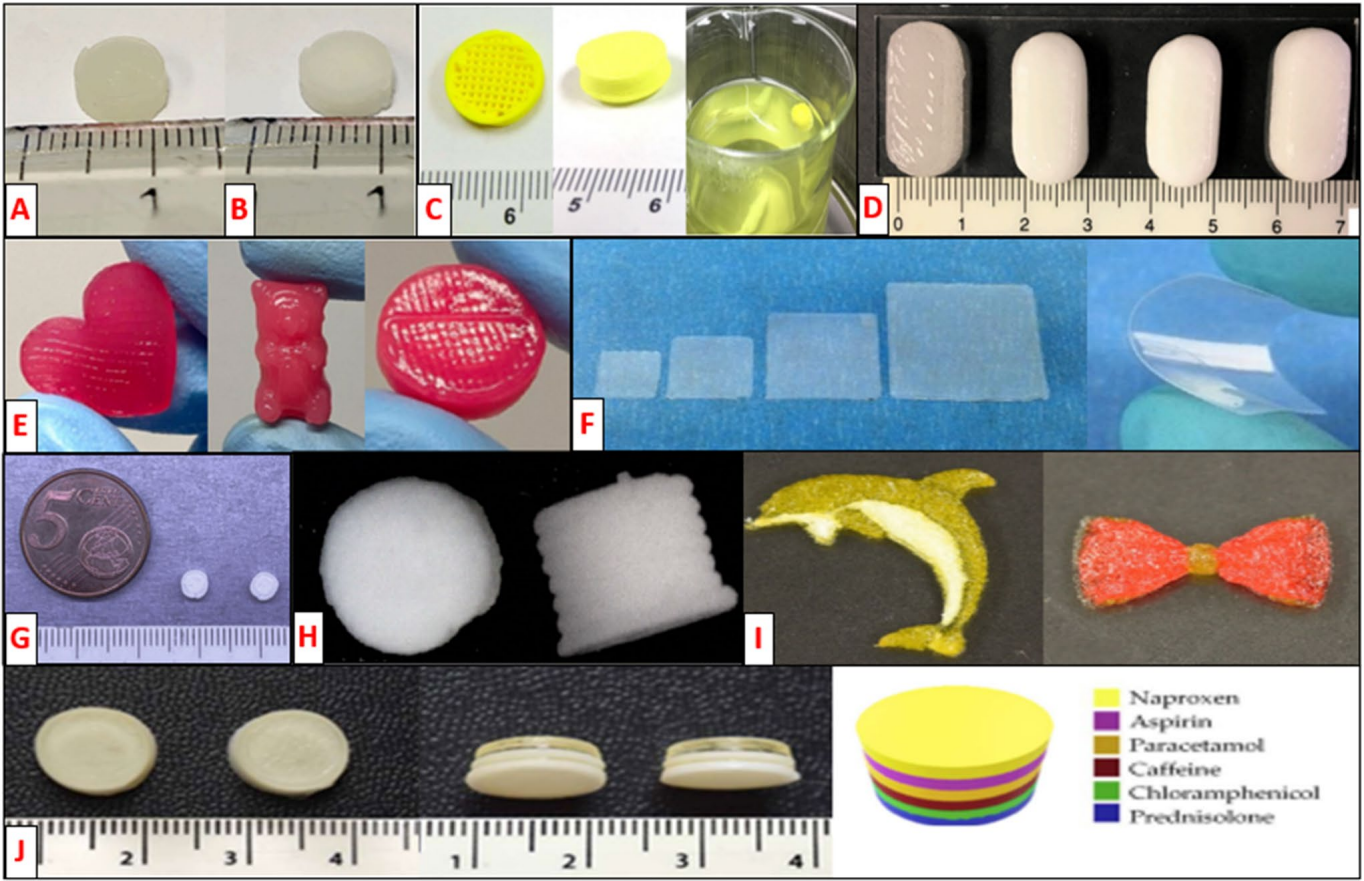
Examples of 3D printed oral dosages forms: (**A**,**B**) furosemide and sildenafil printlets [20]. (**C**) Dipyridamole gastro-floating controlled-release system [21]. (**D**) Extended-release 3D tablets of theophylline [22]. (**E**) Ranitidine chewable printlets [25]. (**F**) Orodispersible warfarin films [27]. (**G**) Hydroclorothiazide micro-extruded orodispersible printlet [26]. (**H**) Carbamazepine orodispersible mini-tablets (diameter ≈ 3 mm, height ≈ 2,2 mm and weight ≈ 10 mg) [29]. (**I**) 3D printed complex shapes food using gelatin B, whey protein isolate, and xanthan gum [34]. (**J**) Multi-layered polypill containing six drugs [31]. Adapted with permission from [20–22, 25–27, 29, 31, 34]

Three-dimensional printing (3DP) of medicines is emerging as a new disruptive compounding technology for the fabrication of a wide range of personalized dosage forms [19] such as printlets (3D printed tablets) [20], extended and controlled-release systems [21, 22], chewable formulations [23–25], orodispersible films [26, 27], minitablets [28, 29], polypills (combination of multiple drugs in the same 3D printed dosage form) [30–32] as well as tailored food products for specific needs [33, 34] (Fig. 1). In comparison with conventional time-consuming compounding methods, 3DP can quickly and accurately produce patient-customized single or multiple-dose pharmaceutical forms with different shapes, sensory characteristics (colors and flavors), sizes and drug release profiles at the point-of-care, such as hospital or community pharmacies [35–37]. The application of this technology is particularly beneficial for patients facing challenges in medication adherence or tolerance, when commercially available dosages are not suitable, and even in situations involving frequent dose adjustments.

Preclinical and even clinical studies of 3DP of medicines have shown promising results at an early stage [38]. Indeed, a study involving 12 healthy adults demonstrated pharmacokinetic bioequivalence between commercial tablets and a 3D printed sildenafil formulation [39]. Recently, the disintegration of 3D printed placebo tablets, prepared by selective laser sintering, was evaluated for the first time in six human volunteers using magnetic resonance imaging [40]. Nevertheless, clinical studies involving personalized printed medicines at the point-of-care remain quite sparse.

The first clinical study was conducted in paediatric patients with a rare metabolic disorder (maple syrup urine disease, MSUD) and demonstrated the acceptability and efficacy of 3D printed chewable tablets, indicating that 3DP offers a viable approach to manufacture personalized medicines on-demand [41]. Additionally, 3DP technology was evaluated as an alternative method to avoid the manual subdivision of levothyroxine sodium tablets in 91 infants with transient hypothyroxinemia [42]. More recently, in the same realm of rare metabolic diseases, a clinical study demonstrated the benefits of preparing chewable printlets containing different amino acids for paediatric patients. For the first time, 3DP enabled the combination of two different treatments into a single chewable printlet, reducing the number of administrations and improving the quality of life of children affected with rare diseases. The study demonstrated that manufacturing and utilizing a dual-component printlet is viable in clinical settings [43]. These studies emphasize that point-of-care 3DP of medicines is becoming a reality in the clinical practice. However, to fully demonstrate this capacity, it is imperative to reinforce these findings with additional clinical studies, particularly in the paediatric population, where the current research is notably scarce. Ongoing research is crucial to substantiate the clinical benefits and enhanced patient acceptability associated with 3D printed pharmaceuticals.

The planning and executing clinical trials with (paediatric) patients involving 3D technologies present significant challenges within both the Spanish and global healthcare landscapes. These challenges arise from the need to navigate complex regulatory frameworks for clinical trials involving medicines, while also ensuring that ethical and legal considerations for participants are fully addressed [44–46] and adhering to the in-coming regulations focused on point-of-care 3DP of medicines [47]. Indeed, there is a lack of an established regulatory framework focused on 3D printing and it depends on the country; however, medicines regulatory agencies like the U.S. Food Drug Administration (FDA) and the U.K. Medicines and Healthcare products Regulatory Agency (MHRA) are adapting regulations to support point-of-care manufacturing [48, 49]. The FDA is exploring regulatory strategies to safely implement point-of-care 3DP, emphasizing on quality assurance, validation, and oversight. Through collaboration with industry and healthcare stakeholders, FDA aims to establish a flexible yet robust framework that encourages innovation while prioritizing patient safety. In the United Kingdom, the MHRA has introduced *The Human Medicines (Amendment) (Modular Manufacture and Point of Care) Regulations 2025*, a new regulatory framework set to come into effect in July 2025 [50]. This framework supports point-of-care (PoC) manufacturing by ensuring safety and quality in the production of innovative, patient-specific medicines at or near the site of treatment. Modular manufacturing—using standardized, portable units—adds flexibility and scalability, making it ideal for producing small batches and personalized formulations within decentralized healthcare settings. In the European Union, the EMA has established the Quality Innovation Group (QIG) to support the integration of advanced manufacturing technologies, including 3D printing, into the regulatory framework. The QIG facilitates early dialogue between developers and regulators, offering guidance on quality-related innovations. Its 2025–2027 work plan emphasizes personalized medicine and includes the development of a ‘Questions and Answers’ document on 3D printing and decentralized manufacturing, aiming to clarify regulatory expectations and support compliance with GMP and quality standards [51–53].

In particular, there is a crucial need to take into account the vulnerability of children, particularly as a protected group in clinical trials, since children have the same rights as adults to be treated [45].

In response to this growing need, Vall d’Hebron Barcelona Hospital is leading the launch of a clinical trial, recently approved by regulatory authorities, to assess the feasibility of implementing point-of-care 3D printed medicines for paediatric patients (EudraCT number: 2021-001069-20, EUCT Number: 2024-519149-31-00 and ClinicalTrials.gov ID: NCT06435481 [54]). Therefore, this article evaluates the feasibility of implementing 3DP for personalised drug manufacture within hospital setting. Additionally, this work can serve as a guide to obtain the clinical trial approval by the Spanish Agency for Medicine and Health Products (AEMPS) to manufacture personalised 3D printed medicines in Spain at the point-of-care. With the aim of reaching international harmonization, this article may serve also as a basis for the implementation in other countries.

## The problem with standard treatments

In the field of pharmaceutical development, creating medications suitable for all demographics presents a constant challenge. Commercial medication generally meets the needs of adult populations, with tablets and capsules being the most used dosage forms. However, deficiencies become evident at the extremes of age brackets since they are often excluded from clinical trials [55]. Elderly individuals often require nuanced dosages compared to adults, particularly those with dysphagia, where mixing thickeners with medicines can affect drug bioavailability, rendering conventional formulations inefficient in meeting their therapeutic needs [56]. Moreover, geriatric populations are affected with different diseases at the same time, and more than one medication needs to be administered to control their afflictions. This issue is especially acute when it comes to paediatric patients, whose distinct physiological and developmental characteristics mandate medications tailored specifically to each child’s requirements [57]. As highlighted by the WHO and EMA, less than a third of developed and tested drugs come in an appropriate form for children leading to a very high prescription rate of off-label medication. Consequently, many children are treated using estimations involving fractions of adult doses, with the medication for adults being manipulated to reach this personalized dose, often in an unautomated fashion taking up substantial time. For example, crushing tablets or extracting part of the contents of a capsule by hand, using medicines that are effectively unlicensed for children [5]. This problem is underscored by the fact that medication errors are three times more common in children than in adults and adverse drug reactions are mostly associated with off-label drug prescriptions [58]. Therefore, the absence of appropriate medication for these populations underscores the need for methods that allow for the safe and timely personalization of dose.

Conventionally, medicines for paediatric patients have been prepared in pharmacies through compounding. This practice, while essential for providing personalized treatment, is not without its drawbacks. Compounding practices, as mentioned above, are often manually carried out, manipulating commercialized medication or hand filling capsules from scratch. These practices lead to increased risks of inaccuracies in dose, contamination and pharmacist exposure to harmful compounds [14, 59]. Compounded formulations also often struggle with issues such as unpalatable taste (some drugs exhibited unpleasant taste), inconvenient liquid formulations due to stability issues, and other factors that contribute to poor adherence such as the difficulty of managing treatment during school hours [15, 16, 60]. Consequently, there are instances of hospital admissions directly linked to non-adherence or incorrect dosages of compounded medications [15]. In fact, 35% of non-adherence to medication in paediatrics is directly related to compounded medication dosage forms [61]. This highlights the importance of addressing the limitations of traditional compounding methods and exploring alternative approaches to medication preparation. To address these challenges, a promising solution lies in incorporating the innovative approach of 3DP into pharmaceutical compounding workflows. This can ensure precise dosing in a user-friendly dosage form, while also automating the preparation process to minimize the risks associated with compounding. Once the 3D printed medication is ready, 3DP technologies could also help to improve medication adherence by creating customized pillboxes tailored to the specific needs of each patient [62].

## 3D printing of medicines

3D printing, also known as additive manufacturing, has been evaluated and used for many years in other sectors such as automotive and aerospace. However, its use in the pharmaceutical field is relatively recent. 3DP of medicines is a flexible manufacturing process capable of creating tailored medications with unique attributes [63]. This process involves the layer-by-layer deposition of drug-loaded materials (pharma-inks) based on a digital-aided design software, enabling the creation of customized medication formulations [19].

**Fig. 2:**
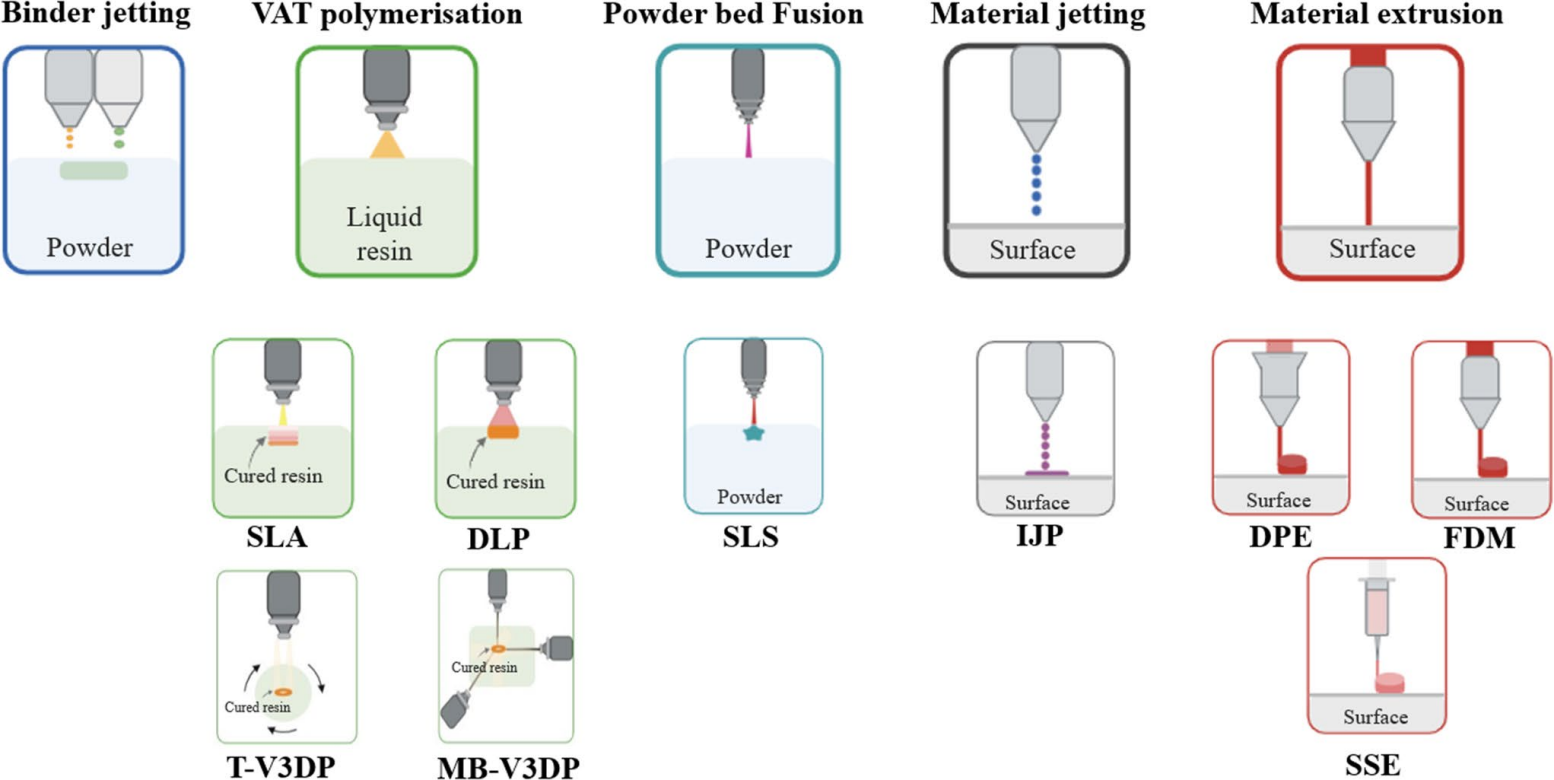
Graphical representation of the different 3D printing methodologies utilized in the pharmaceutical sector. Binder jetting; VAT polymerization (stereolithography (SLA), direct light processing (DLP), tomographic volumetric 3D printing (T-V3DP) and multibeam volumetric 3D printing (MB-V3DP); powder bed fusion (selective laser sintering, SLS); material jetting (Inkjet printing, IJP); material extrusion options include fused deposition modelling (FDM), semi-solid extrusion (SSE) and direct powder extrusion (DPE). Adapted from [37]. Figure created with BioRender.com.

According to the American Society for Testing and Materials (ASTM) there are seven main 3DP technology categories, each with its own unique approach and applications [64]. In the pharmaceutical sector, many of these are commonly utilized in research, namely, binder jetting, VAT polymerisation, powder bed fusion, material jetting and material extrusion (Fig. 2) [19, 63, 65, 66]: (1) Binder jetting entails the selective deposition of a liquid binding solution, using a printer nozzle, onto a powder bed in which wetted powder particles adhere together to create solid layers. (2) VAT polymerization involves curing liquid resin with a light source (a laser or projected light), causing solidification by photopolymerization. This process comprises of different technologies, with stereolithography (SLA) and digital light processing (DLP) being the most used for pharmaceutical purposes. Additionally, a novel VAT photopolymerization technique called volumetric 3D printing, with two different approaches (tomographic and multi-beam) promises higher printing speeds and improved mechanical properties. (3) Powder bed fusion employs a laser or electron beam to selectively fuse powdered material together to form the 3D object, layer by layer, for example selective laser sintering (SLS). (4) Material jetting works by deposition liquid droplets of material onto a working surface, which are then cured or spontaneously solidified, for example with inkjet printing (IJP). (5) Material extrusion, perhaps the most promising method for manufacturing small batches and personalized medicines, involves extruding material through a nozzle to create an object layer by layer. This last 3DP process includes methods such as fused deposition modelling (FDM), in which a polymer filament is heated and extruded; direct powder extrusion (DPE), in which powdered material is heated and extruded through a heated printhead nozzle; and semi-solid extrusion (SSE), based on the extrusion of a gel, waxes or paste within a syringe-like system. The latter also being usable to fill capsules, molds or blisters directly as an automated, computer-aided filling process for pharmaceutics.

Some of the 3D printing technologies mentioned have already been implemented for mass manufacturing of medicines. Aprecia Pharmaceuticals in the US made a significant breakthrough by adapting the binder jetting process into a mass manufacturing process in 2015 [67]. This innovation, termed ZipDose technology, paved the way for the first 3D printed tablet (Spritam^®^) approved by the FDA for clinical use. Although Spritam^®^ (which contains the antiepileptic medication levetiracetam) marked a milestone in pharmaceutical manufacturing, it was not designed for personalized treatment and offer only four fixed doses (250 mg, 500 mg, 750 mg and 1000 mg). Similarly, Triastek, a pioneering pharmaceutical company specializing in 3DP based in China, has developed its proprietary 3DP process called “*melt extrusion deposition*” (MED^®^) [68, 69]. This technology enables the mass-production of pharmaceuticals using API and excipients materials in powder form as starting materials. It allows for the creation of a wide range of structural designs to achieve the desired release profile, thereby improving the effectiveness, safety, and overall patient experience with medication therapy. Through the utilization of MED^®^, Triastek aims to address critical challenges in drug formulation and delivery, ultimately improving the pharmaceutical industry’s approach to mass manufacturing. To date, Triastek has four pharmaceutical products in its pipeline that are in the early clinical phase [68].

Despite their focus on large-scale production of 3D printed medications and the numerous benefits associated with this technology, both companies are neglecting a significant advantage offered by 3DP: the ability to prepare on-demand customized medications tailored to the unique needs of individual patients in a decentralized environment (hospitals or community pharmacies). In light of this latter advantage, extrusion-based 3DP is emerging as a new technique capable of revolutionizing conventional pharmaceutical compounding at the point-of-care [39, 41, 70] for multiple patient groups, including paediatrics allowing for precise dosage adjustments and the creation of child-friendly dosage forms [19, 41, 71].

In the context of personalization, the advantages of 3DP in the pharmaceutical field can be leveraged not only to enable the accurate dosages of medications, but also to allow the combinations of multiple drugs (polypills), the modification of release patterns, and the customization of color, flavor and shape of medicines according to the child’s preferences to improve treatment adherence [19, 41]. Furthermore, a unique benefit of 3DP that is not possible with other manufacturing methods (such as standard pharmaceutical compounding) is the inclusion of codes or patterns on the surface of the printlets for identification or tracking purposes. Indeed, printlets with Braille and Moon patterns suitable for visually impaired patients have been developed [72], as well as orodispersible films with quick response (QR) codes or data matrices encoding relevant information such as the required dose, patient’s details, posology, route of administration and batch number [73]. These characteristics can greatly enhance medication adherence and safety in children and reduce the resistance they may have towards taking medication [16, 74].

## 3DP at the point-of-care

There is considerable interest in the development and testing of 3DP technologies for the preparation of personalized medicines at the point-of-care. As previously reviewed by Seoane-Viaño et al. [19], this innovative technology enables the creation of medicines in wide array of shapes and sizes, tailored to patient’s needs with precise dosages for both preclinical and clinical use. The automation speeds up compounding processes, reduces involved manual labour [75] and risks associated with standard compounding practices, for example contamination, inaccurate dose and pharmacist drug exposure [14]. Moreover, its affordability facilitates its deployment in research labs and hospitals. Over the years, new advancements have emerged that are further adapting this technology for application point-of-care clinical settings. A selection of articles evaluating the implementation of pharmaceutical 3D printing in hospitals and pharmacies that also consider the regulatory requirements are included in Table 1.

**Table 1:** A selection of articles evaluating the implementation of 3D printing of medicines in hospitals and pharmacies which also consider the regulatory requirements

Type of study	Publication year	Location	Population	Disease	Drug and dose	Reference
Acceptability study	2017	University College London, United Kingdom	Healthy volunteers aged 18–45 years (*n* = 50)	-	Placebo	[76]
Clinical study	2019	Clinical Hospital of Santiago de Compostela, Spain	Paediatric patients aged 3–16 years (*n* = 4)	MSUD	Isoleucine (50–150 mg)	[41]
Acceptability study	2020	East London primary school, United Kingdom	Healthy children aged 4–11 years (*n* = 368)	-	Placebo	[77]
Characterization study and clinical study	2020	Guangdong Provincial People’s Hospital, China	Inborn patients aged 1 day to 9 months (*n* = 11)	Not reported	Spironolactone (2 mg)	[71]
Clinical study	2023	Guangdong Women and Child Health Hospital, Shunde, China Women Children’s Hospital of Guangdong Medical University, China	Premature infants aged 0–5 years (*n* = 91)	Transient hypothyroxinemia	Levothyroxine sodium (5, 10, 12.5, 16.7 and 20 µg)	[42]
Bioequivalence study	2023	Leiden University Medical Center, The Netherlands	Healthy volunteers aged 18–55 years (*n* = 12)	Pulmonary arterial hypertension	Sildenafil citrate (10 mg)	[39]
Future clinical study	2023	University Medical Centre Hamburg-Eppendorf, Germany	Adult patients (*n* = 50)	Parkinson’s disease	Levodopa/carbidopa	[70]
Characterization study	2023	University Medical Centre Hamburg-Eppendorf, Germany	-	Parkinson’s disease	Levodopa/carbidopa (60/15 mg and 170/42.5 mg)	[78]
Characterization study	2023	Erasmus University Medical Center, The Netherlands	-	Adrenal insufficiency	Hydrocortisone (2, 4, 6, 8, 10 and 12 mg)	[79]
Clinical study	2024	University Hospital Lucus Augusti, Spain	Healthy adult volunteers (age not reported) (*n* = 6)	-	Placebo	[40]
Clinical study	2024	Clinical Hospital of Santiago de Compostela, Spain	Paediatric patients aged 6–14 years (*n* = 6)	MSUD, ECHS1 deficiency and OTC deficiency	Isoleucine (600 and 650 mg) Valine (500 mg) Combination of isoleucine (200 and 450 mg) and valine (200 and 350 mg) Citrulline (600 and 950 mg)	[43]
Characterization study	2024	Gustave Roussy, France	-	Breast cancer	Tamoxifen citrate (20 mg) Duloxetine (pellets, 37.5 and 75 mg) Venlafaxine (pellets, 30 and 60 mg)	[80]
Characterization study	2024	Vall d’Hebron Barcelona Hospital, Spain	-	Adrenal insufficiency	Hydrocortisone base (1, 2, 3, 4, 5, and 6 mg)	[81]
First clinical implementation in a Pharmacy	2025	Ángel Orive compounding pharmacy, Spain	Adult patients	Alopecia	Minoxidil (2.5 and 5 mg)	[75]

The first study to evaluate the acceptability of 3DP started in 2017 using FDM printing technology [76]. This early exploration into the acceptability of 3D printed medicines revealed significant potential for patient-centered approaches in pharmaceutical formulations. The study demonstrated that the physical characteristics of 3D printed tablets (shape, size, and color) influence patient acceptance. Shapes resembling conventional dosage forms, such as capsules and discs, were readily accepted by patients, indicating that familiarity plays a crucial role in patient compliance. In another study, four different 3D printing technologies (DLP, SLS, SSE, and FDM) were used to create placebo printlets with similar physical attributes such as size and shape [77]. Through a questionnaire administered to 368 participants aged 4–11 years, the DLP printlets were selected as the most visually appealing. However, when participants learned that SSE printlets were chewable, they favoured these over their initial choices. These studies highlight the importance of considering end-user preferences in the design of new pharmaceutical products, emphasizing the advantage offered by 3DP technology in adapting flexibly to these preferences, thereby potentially improving adherence.

Further advancing the application of 3DP in pharmacy, in 2019, a subsequent study by Goyanes et al. focused on the preparation of isoleucine for the treatment of Maple Syrup Urine Disorder (MSUD, a rare metabolic disorder) [41]. The treatment of metabolic disorders such as this involves dietary protein restriction and amino acid supplementation. This specific study explored the use of 3DP technology to create a personalized formulation of isoleucine, which is essential for managing the amino acid metabolism in MSUD. The study demonstrated that 3DP can accurately and reproducibly create tailored printlets with different doses, colors and flavors, offering a novel method of preparing individualized therapeutic options. This study was the first one to demonstrate that medicines produced by 3D printing were as effective as medicines produced by pharmaceutical compounding at the hospital setting but with greater acceptability than conventional treatment. The use of 3DP for such applications not only enhances the precision of the dosage but also enables the rapid production of treatment formulation that are well accepted and effective in the management of MSUD.

One year later, the application of 3DP technology for the preparation of subdivided printlets of hydrochlorothiazide for precise hospital dispensing was evaluated [71]. This study demonstrated that 3DP could be effectively used to create subdivided tablets (with predefined fracture lines) that maintain accurate dose divisions. Years later, the same team undertook a pivotal study aimed at addressing the challenges of dosing levothyroxine in hormone replacement therapy for transient thyroid disorders in premature infants using printlets of levothyroxine [42]. The findings revealed that these subdivided printlets exhibited enhanced uniformity in drug distribution and dose accuracy compared to commercially available levothyroxine tablets. This contribution to the field of 3D printed pharmaceuticals reinforces the versatility and utility of this technology in clinical applications.

More recently, Lyousoufi et al. evaluated the bioequivalence of printlets prepared compared to a commercial formulation [39]. They selected sildenafil as a model drug and successfully demonstrated, through a detailed pharmacokinetic study, the potential of this technology in creating tailor-made drug formulations that are bioequivalent to the commercially available sildenafil tablets (Revatio^®^). The findings confirmed the technical feasibility of producing bioequivalent 3D printed medications in a hospital setting.

In addition, significant progress was made in the field of patient-specific 3DP by a research team in Germany. First, the study of Langebrake et al. explored the integration of 3D drug printing in hospital pharmacies with a machine-learning-assisted closed-loop system to dynamically optimize dosages [70]. This system would utilize real-time data from smartwatches worn by patients to adjust the required drug dose in the printlets, enhancing treatment precision by adapting the medication to the patient’s current health status. In a separate publication, the same team detailed the manufacturing processes they developed and achieved pharmaceutical validation levodopa-containing printlets they prepared [78]. Employing DPE, they developed levodopa printlets with high dose accuracy, high crushing strength, very low friability and immediate drug release, utilizing a specific excipient. With these results, they demonstrated that DPE is suitable for implementation in closed-loop medication management systems in hospital pharmacies.

In 2024, the field saw exciting new innovations, one of the standout studies being conducted by Seoane-Viaño et al. [40]. Their research explored the journey of placebo printlets through the gastrointestinal tract, offering a groundbreaking approach using magnetic resonance imaging to provide real-time visualization of printlet disintegration in human volunteers. This study significantly advances our understanding of pharmacokinetics, ensuring that 3D printed drugs are released and dissolved at precise rates and targeted locations within the GI tract. By comparing in vitro and in vivo performance, it paves the way for more effective and personalized drug delivery. Another contribution from the same group explored the feasibility of combining two distinct amino acids within a single chewable printlet for the effective management of rare metabolic disorders in paediatric patients [43]. Their findings booster previous publications reporting that printlets do not only exhibit less deviations of amino acid blood levels but also enhance patient acceptability and adherence to the treatment regimen. Additionally, in this study, the authors implemented the use of a mobile app (M3DIFEEDBACK) by the patients to obtain acceptability feedback in real-time. This study emphasizes the practicality of using 3D printing technologies to manufacture polypills, showcasing its transformative impact on current pharmaceutical practices.

Advancing polypill research and development, clinical researchers at Gustave Roussy Institute, Europe’s leading oncology hospital based in Paris, are conducting a study focused on integrating multi-drug 3D printed formulations into clinical practice. Their initial report details the development of the first formulations and validation testing, setting the stage for upcoming clinical evaluations [80]. This research focuses on the development, printing and clinical application of personalized polypill that combines anticancer therapy (tamoxifen) with medication to manage side effects (venlafaxine or duloxetine), specifically for the treatment of early-stage breast cancer patients. They used a pharmaceutical 3D printer M3DIMAKER with a production rate of 200 dosages units per hour. The printer filled capsules with a semi-solid tamoxifen pharma-ink using a SSE printhead, followed by the dispensing of either commercially-available venlafaxine or duloxetine pellets through a novel pellet dispensing printhead [80].

To promote the implementation of 3DP at the point-of-care more studies and collaborations are necessary. The International Pharmaceutical 3DP Initiative (Pharma3DPI) was recently established with the aim of enhancing the use of 3DP technology in the pharmaceutical sector to advance patient-centric healthcare solutions, specially hospital settings [82]. The Pharma3DPI consortium brings together a diverse group of members from academia, healthcare, pharmaceutical industry, and regulatory bodies. This collaboration seeks to facilitate the exploration of emerging technologies, which could lead to the development of personalized medicine and offer significant benefits to patients worldwide.

As we look to the future, the trajectory of 3DP in pharmaceuticals points toward an era where drug formulation is not only personalized but also optimized based on the patient’s needs. Its integration into clinical settings, such as hospitals, will ensure that treatments are not only effective, safer and more acceptable, but also closely aligned with the metabolic and physiological characteristics or preferences of individual patients.

Recent MHRA regulations in the UK will established a framework for Modular Manufacture and Point of Care, enabling the on-demand manufacturing of patient-specific treatments, such as 3DP medicines, within healthcare facilities [50]. This approach will be especially beneficial for conditions requiring precise dosing, rapid formulation adjustments, or short-shelf-life medications. As regulations evolve, point-of-care production is expected to further bridge the gap between pharmaceutical innovation and personalized medicine [83].

We also look forward to the outcomes of the ongoing clinical study at Vall d’Hebron Barcelona Hospital (EudraCT number: 2021-001069-20, EUCT Number: 2024-519149-31-00 and ClinicalTrials.gov ID: NCT06435481 [54], specifically designed for paediatric patients with adrenal insufficiency undergoing hydrocortisone treatment. This study, already approved by the regulatory authorities in Spain (AEMPS), is expected to support the integration of 3D printing technologies into drug formulation processes directly at the point-of-care in hospital settings, ultimately expanding the reach and impact of personalized pharmaceutical solutions.

## How to plan and develop 3D printing clinical trials

3D printers can be integrated into daily clinical practice, such as in pharmacies or hospitals, in accordance with compounding regulations in most countries [75]. This approach would enable treatment of patients, but it may not provide the means to assess clinical outcomes outside conventional clinical practice. As a result, it is crucial to continue conducting clinical trials to gather data and demonstrate the true benefits of 3D printing in personalized medicine.

The first step for planning any type of clinical trial is to understand the regulatory framework governing clinical trials involving human subjects. In the European Union, this information can be found on the EMA website [84]. For Spain, more specific information is available on the AEMPS website [85]. A crucial document that serves as a guide to fulfill all necessary information and procedures for planning and initiating a clinical trial is the “Instruction Document of the Spanish Agency of Medicines and Medical Devices for Conducting Clinical Trials in Spain” [86] and its annexes [87], available on the AEMPS website [88].

The planning and development of clinical trials involving 3DP present several challenges and obstacles that need to be carefully addressed. These challenges are mainly related to regulatory barriers such as obtaining ethical approval [46], following regulatory requirements [44], recruitment of patients [89] and patient compliance [45]. It is equally important to factor in the costs and timeframes involved in clinical trial development. Furthermore, it should be noted that long lead times from protocol approval to trial activation may be due to administrative burdens imposed by external laws and regulations or internal institutional requirements imposed by the site where the trial will be conducted [90].

The challenges of setting up a clinical trial increase particularly with paediatric patients, considering their unique characteristics: the ongoing growth and development of these patients, including their organ maturation, cognitive development, and unique pharmacokinetics and pharmacodynamics, necessitate careful consideration. Moreover, children, due to their age, lack the capacity to provide informed consent independently. As a result, it is essential to obtain consent from a parent or legal guardian to ensure ethical and legal compliance [5, 6, 44, 45].

These challenges are magnified when incorporating a new manufacturing technology that has only been employed in a few published works worldwide [41, 43], with current use in standard compounding workflows not being easily publishable and shared under the associated regulation. The integration of new manufacturing technologies, such as 3DP, introduces additional complexities into clinical trial planning and execution. Unlike traditional compounding/manufacturing methods, 3DP requires specialized equipment (3D printer), expertise, and validation processes. Incorporating these elements into clinical trial protocols demands careful consideration to ensure consistency, reproducibility, and regulatory compliance across different trial sites and batches of medications.

Mirroring new formulations prepared using standard techniques, medicines manufactured using 3DP technology must undergo the optimisation and validation of the entire preparation (including printing) process to ensure the quality of the resulting printed product before it is given to patients as part of clinical trials or regular compounding workflows. This involves evaluating factors, such as the physical and chemical characterization of the pharma-ink and the printed medicine, using in vitro tests including dissolution and organoleptic characteristics, along with a full explanation of the elaboration process.

**Fig. 3:**
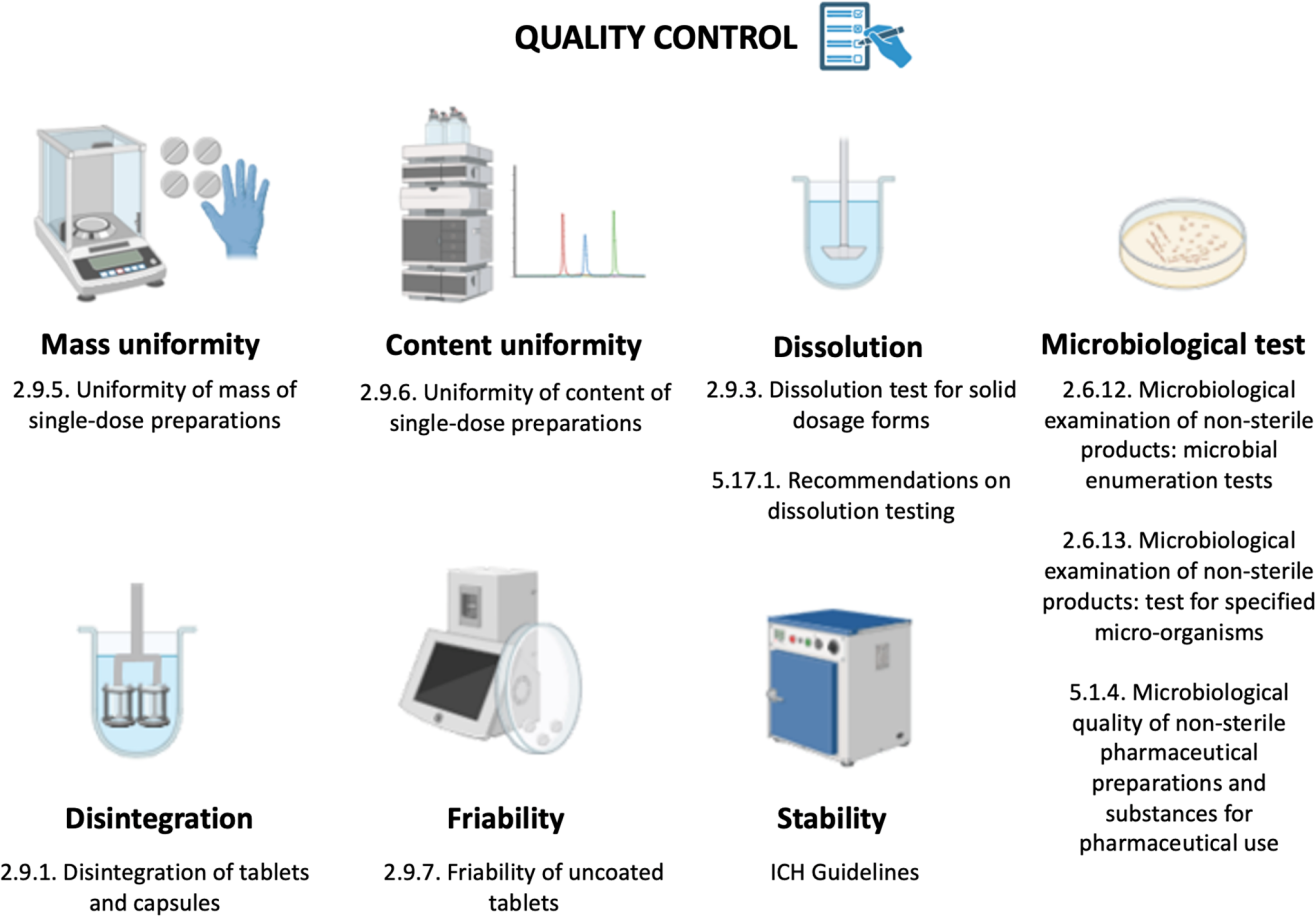
Main quality control tests applicable to solid printlets according to Ph. Eur

The goal of the validation step is to demonstrate that the resulting new formulation consistently meets the predetermined Critical Quality Attributes (CQAs) in real-world scenarios. A detailed explanation of the validation of printing process for submission to regulatory authorities for clinical trials can be found in a previous article published by our team. The manufactured drug undergoes evaluation to ensure compliance with the quality standards delineated in the European Pharmacopoeia (Ph. Eur.) 11th edition. Thus, multiple sets of medicines with varying dosages must be examined to assess their properties and ensure they meet pharmacopeia quality criteria [81]. Since printlets are a solid pharmaceutical form, tests applicable to solid forms may include: (1) disintegration of tablets and capsules (Ph. Eur. 2.9.1.), (2) dissolution test for solid dosage forms (Ph. Eur. 2.9.3.), (3) friability of uncoated tablets (Ph. Eur. 2.9.7), (4) uniformity of mass of single-dose preparations (Ph. Eur. 2.9.5.), (5) uniformity of content of single-dose preparations (Ph. Eur. 2.9.6), and (6) microbiological quality of non-sterile pharmaceutical preparations and substances for pharmaceutical use (Ph. Eur. 5.1.4). Tests applicable to printlets with the corresponding Ph. Eur. monographs are summarized in Fig. 3.

Although these tests are compulsory, they may not be the best suited for this new type of manufacturing. This quality by test approach is unsuitable in 3D printing as the aim is to produce small batches of personalised medicines on demand and in many cases would require the entire batch or the manufacturing of additional units to carry out assays that are mandatory by Ph. Eur. Therefore, tools based on process analytical technologies (PAT) are being installed in-line, on-line, at-line or off-line into 3D printers to allow critical quality attributes to be identified, preventing the destruction of the 3D medicines manufactured. Examples of PAT tools that were successfully investigated consisted of a pressure sensor for rheological characterization [91], near-infrared spectroscopy for content uniformity test [92–95] and an in-line and software-assisted balance for mass uniformity testing [96, 97].

To ensure the long-term integrity and the efficacy of the printlets, and assess how it holds up under different storage conditions (packing, temperature, humidity and light) stability studies must be conducted following guidelines established by the International Council for Harmonisation of Technical Requirements for Pharmaceuticals for Human Use (ICH) [98]. Additionally, due to the thermal stress involved in the 3DP process, thermoanalytical tests are desirable to assess potential degradation, presence of impurities or alteration in the active ingredients during manufacturing. Finally, conducting bioequivalence evaluations could provide crucial insights into how well the printlets align with the conventional treatments. However, it is important to recognize that 3D printed medications are inherently personalized and specifically tailored to meet individual patient needs. As a result, they cannot be directly compared to standardized, commercially available drugs with equivalent doses, since such reference formulations often do not exist. This fundamental absence of a suitable comparator renders traditional bioequivalence studies impractical in most cases. Moreover, regulatory frameworks provide biowaivers for certain drugs, allowing them to proceed to clinical use without requiring bioequivalence studies, particularly when supported by other forms of evidence, like in vitro dissolution testing.

The central aim of our clinical study is therefore not to demonstrate equivalence with existing medications, but to evaluate the efficacy and safety of these customized treatments in real-world settings. In this context, clinical outcomes become the key measure of therapeutic performance, making conventional bioequivalence assessments largely irrelevant. It is quite clear that regulations for medicines are not designed for the incorporation of 3DP technologies within clinical trial settings, but the research focused on this novel technology is advancing rapidly.

## Considerations for clinical trial approval involving 3DP

This section reviews the key aspects to consider when applying for a clinical trial using 3DP, following a chronological order. The authors developed the general points to follow based on personal experience with the Spanish regulatory agency (AEMPS) (Table 2).

**Table 2:** Key points and choices to consider for clinical trial approval. BCS: biopharmaceutics classification system; 3DP: Three-dimensional printing and SOP: standard operating procedure

Selecting a suitable disease and drugs for clinical trial development	Disease requiring continuous dose adjustment. Disease treatment dependent on compounding. Treatment adherence problems. Chronic treatment. Therapeutic drug monitoring. Pharmaceutical aspects (81): (1) Printing methods published, (2) BCS, (3) Availability of high-performance liquid chromatography, (4) stability, and (5) temperature of degradation.
Patient recruitment, characteristics and requirements	Sufficient patients affected by the disease to be able to recruit. Able to follow the treatment. Age-appropriate informed consent, assent processes and involvement of parents. Tailoring recruitment strategies. Potential impact on their daily lives. Adherence safety protocols. Monitoring for potential adverse events. Meet strict ethical standards. Possible use of mobile applications for patient tracking and patient information sharing. Compliance with Good Clinical Practice
Equipment, facilities and printing technology for compounding	3D printer available for decentralised production. 3D printer with an integrated balance. 3D printer with associated software specifically for use in pharmaceutics Pharmaceutical Technology. Good Manufacturing Practice facility. Physicochemical properties of the selected drugs. Facility requirements: microbiological controls, attire, hygiene, cleanliness, and air quality.
Regulatory requirements for the clinical trial	Audit of the facilities. Pharmaceutical validation compliance. Creation of a dossier and patient information sheet and consent. Regulatory feedback. Creating of SOP for: (1) 3DP medication fabrication, (2) 3D printer usage log, (3) Procedure for release batches, (4) Facilities and equipment cleaning, (5) Personal formation, (6) Staff organization chart, (7) Incident management procedure, (8) Reading records, (9) Procedure for returns and withdrawals, (10) Complaints procedure, and 11) Clinical trial documentation retention.

### Selecting a suitable disease and drug/s for clinical trial development using 3DP

Among the diseases affecting the paediatric population, it is essential to focus on those lacking specifically marketed drugs for this demographic or where medications marketed are not well-suited for all patients, especially children. Critical aspects to consider involve constant dosage adjustments based on current symptom control or the measurement of biomarkers. Within these medications and from a clinical perspective, it is interesting to choose those prescribed chronically and where literature or clinical practice has demonstrated poor therapeutic compliance due to difficulties in dosing or palatability. With 3DP, this last issue can be overcome by producing chewable medicines with different flavors and colors [23–25, 76]. Notably, the ability to measure drug plasma levels or parameters guiding effectiveness is crucial, leading us to medications requiring constant dose adjustments or administration frequency changes.

Regarding these points, adrenal insufficiency pathology, which affects either the adrenal glands or the pituitary gland resulting in deregulation of cortisol or adrenocorticotropic hormone synthesis, shows potential as a candidate disease for 3DP to target. It is diagnosed in childhood with a prevalence of approximately 150–280 cases/million, as a chronic disorder patients need to be treated throughout their lives. The standard treatment for all causes of adrenal insufficiency involves cortisol replacement therapy, with hydrocortisone administered chronically to children at doses ranging from 8 to 12 mg/m^2^/day, administered two to four times daily [99]. Few hydrocortisone oral commercial products in Spain are available for children and tablets are the most common presentation. However, quartering hydrocortisone tablets can lead to inconsistent cortisol levels, potentially resulting in either undertreatment or overtreatment leading the occurrence of an adrenal crisis, alongside inadequate disease control due to unacceptable dose variability [100, 101]. Consequently, the lack of commercially available hydrocortisone formulations suitable for paediatric patients complicates dosing for children, leading to reduced precision. This gap forces reliance on compounded medications, such as hard capsules or oral suspensions, which come with inherent risks and limitations [102]. For example, the bitter taste of hydrocortisone oral suspension results in poor acceptance of oral hydrocortisone suspension and capsules may not meet Ph. Eur. specifications regarding drug content and mass uniformity [103].

Furthermore, the possibility of assessing treatment effectiveness by measuring clinical parameters (blood pressure, among others) and biochemical markers (cortisol, adrenocorticotropic hormone.) makes this disease an ideal candidate for clinical evaluation [99].

Regarding pharmaceutical aspects and drug selection, the existence of published printing methods, its classification in the Biopharmaceutics Classification System (BCS), the availability of high-performance liquid chromatography (HPLC) methods for pharmaceutical validation, stability, and its decomposition temperature (only if elevated temperatures during printing are required). BCS could be a critical and potentially limiting factor in pharmaceutical development. Depending on the BCS category (Class 1: high solubility– high permeability; Class 2: low solubility– high permeability; Class 3: high solubility– low permeability; Class 4: low solubility– low permeability), predicting the in vivo behavior of the drug can vary. Drugs with low solubility and low permeability (BCS Class 4) are expected to have poor absorption across the intestinal mucosa, resulting in low bioavailability and high variability. Conversely, those with high solubility and high permeability (BCS Class 1) are well absorbed, making these drugs good candidates for 3DP, as is the case with hydrocortisone [104].

### Patient recruitment, characteristics and requirements

Successful recruitment for any clinical trial benefits from a disease/treatment selection which impacts a substantial patient population, whether within the trial centre or at other participating institutions. Considering the printlets as a solid dosage form, assessing patient’s ability to swallow its necessary. EMA recommends the use of solid formulations for children older than 6 years [105].

Ensuring age-appropriate informed consent, assent processes, and active parental involvement in decision-making are essential for patient recruitment. When including paediatric patients, two separate informed consent documents are required: one for parents and another for children, written in clear, age-appropriate language. Children over 12 years old should be able to understand and sign their own assent form. This guarantees a thorough understanding among patients of the research objectives and the significance of their participation in the improvement of their disease management. By engaging participants to clearly understand the research project, their sense of control and involvement is enhanced. Furthermore, recruitment strategies must be adapted effectively for children, adolescents, and families to optimize participation rates. Potential impact on patients’ daily lives, involving changes in routines and added time and effort, should be considered during participation [106].

Strict adherence to ethical and legal standards, coupled with following safety protocols, with careful consideration and monitoring of potential adverse effects, ensures the trial’s scientific integrity while prioritizing paediatric participants’ well-being. As part of this monitoring, any serious and unexpected adverse reactions that occur during the trial must be documented using a designated reporting form and communicated to the relevant drug agency as soon as possible [44].

Using mobile applications installed on patient or parent mobile phones for recording patient acceptability data can streamline collection, enhance data accuracy and timeliness, and improve patient monitoring and management efficiency during the trial [43, 107, 108]. An example is the successful incorporation of the M3DIFEEDBACK mobile app, which allowed patients to provide real-time acceptability feedback [43]. Additionally, mobile applications play a crucial role in improving adherence to treatment regimens by providing reminders, facilitating patient engagement, and enhancing communication with healthcare professionals. Such applications could increase patient convenience and promote better trial engagement by simplifying data reporting and strengthening interactions between patients and researchers [107, 108].

The involvement of the medical team in patient recruitment is a crucial factor in the success of the trial. They ensure patient eligibility, provide therapeutic follow-up, and monitor health throughout the study, managing side effects and adjusting treatments as needed. Their direct involvement helps ensure protocol compliance and enhances the trial´s integrity. Additionally, investing in the training of a research team with the necessary expertise is vital to address the various challenges that may arise during the study [109]. For example, compliance with Good Clinical Practice (GCP) is mandatory for the research team [110]. At the same time, it is important to consider the perspectives of healthcare professionals regarding the potential benefits of 3D printed oral formulations. A survey conducted at a hospital in Singapore found that over 60% of medical doctors would support prescribing 3D printed dosage forms. However, both doctors and pharmacists expressed concerns about the formulation and manufacturing processes, particularly related to quality control, stability and bioequivalence, and administrative issues [111].

### Equipment, facilities and printing technology for compounding

Specialized equipment and facilities are essential. A Good Manufacturing Practice (GMP) room in the hospital pharmacy [112, 113], equipped with state-of-the-art compounding and 3DP technology is required. Ideally, this 3DP equipment should integrate a balance for in-line mass uniformity testing, enhancing quality control [96]. Given that many hospitals may not have a GMP-certified manufacturing room, an alternative would be to produce the medication in another hospital or in an accredited pharmaceutical laboratory and send the medication to the hospital for reconditioning. The facility’s stringent adherence to specific environmental conditions is necessary to guarantee the consistent production of top-tier pharmaceuticals. This includes controlled access facilitated by airlock systems (SAS) to mitigate the risk of personnel-induced contamination, coupled with a continuous supply of filtered air to establish and maintain a cleanroom environment, thereby minimizing the potential for airborne particulate contamination [112, 113].

Maintaining strict control and registration over the pressure, humidity, and temperature as well as regular air qualification assessments, and periodic microbiological controls must be conducted in this area to ensure continuous compliance with established standards and safeguarding the integrity of the pharmaceutical manufacturing process. Furthermore, detailed cleaning and disinfection protocols for the pharmaceutical 3D printer. Specific protocols for cleaning and disinfecting the technology facility and 3DP equipment along with strict protocols governing personnel attire and hygiene are required for maintaining a hygienic manufacturing environment, preventing cross-contamination and ensuring the production of pharmaceuticals with the highest quality standards [112, 113].

The 3D printer utilized in the production of medicines must meet GMP standards and other regulatory requirements for manufacturing medicines within a hospital pharmacy setting [47, 112]. To effectively address the diverse needs of patients and the specific requirements associated with the APIs employed in the preparation of patient-specific drug doses, the printer should possess the capability to fabricate printlets with various materials, utilizing multiple extrusion printing techniques such as SSE, FDM and DPE [114]. The selection of an appropriate printing process is contingent upon the physicochemical properties of the chosen APIs and characteristics of the technique, with different processes proving more suitable for various formulations and manufacturing of specific drugs. Each material extrusion printing technique has its own advantages and disadvantages that must be considered. For instance, SSE allows for the printing at lower temperatures but requires syringable viscosities with rigorous control of extrusion conditions to prevent nozzle clogging. With FDM, the drug release patterns can be easily tuned by varying the infill percentage, but high printing temperatures are needed for extrusion, which poses a risk of drug degradation. DPE involves mixing solid powder APIs and excipients in the printer, thus avoiding the filament production step of FDM, but it presents challenges related to cleaning and preventing cross-contamination [19]. In addition, it is useful to have appropriate healthcare software that allows easy control of the printer and modification of doses to facilitate its use by pharmacists or healthcare personnel. This software must undergo proper validation to ensure that it meets regulatory standards, including verifying its accuracy, reliability, data integrity and consistency in dose preparation and printing operations.

As the EMA considers chewable tablets the preferred formulation for school-age children and adolescents, the 3DP technologies utilized must possess the capability to manufacture chewable tablets with suitable texture, taste, and palatability. The most suitable 3D printing technology that can meet this specific requirement is SSE [23, 24, 41, 43]. However, care must be taken to ensure that the formulation is not overly appealing to children, avoiding candy-like characteristics to mitigate the risk of accidental toxicity [115].

### Regulatory requirements for clinical trials

Creating a comprehensive dossier is the initial step for presenting any clinical trial. This dossier contains detailed information about the trial, including the rationale, objectives, methodology, participant criteria, efficacy and safety assessment, statistical analysis, ethical considerations, data handling, financing, and insurance [116]. It also includes documentation regarding the investigational 3D medication and its commercial comparator, if applicable, including its composition, manufacturing process, and quality control measures [86, 87, 113].

Furthermore, it is important to establish thorough standard operating procedures (SOP) to ensure consistency, quality, and regulatory compliance throughout the trial. Firstly, SOPs are created for both pharma-ink and 3D printed medication fabrication, detailing the manufacturing process, including material selection, printing parameters, post-processing steps, and quality control measures to ensure reproducibility and quality. A separate SOP should be crafted for maintaining a usage log for the 3D printer that records all printer activities, such as start-up and shutdown procedures. Ideally these procedures would be built into the associated pharmaceutical 3DP software. Additionally, SOPs are needed for other formulation fabrication and procedures such as batch releases, personnel training, staff organization, cleaning, among others [87, 113].

Facility audits from the competent authority (in Catalonia the Directorate General of Health Planning and Regulation) constitute another obligatory aspect of regulatory compliance [85, 113], wherein manufacturing facilities undergo rigorous assessments to ensure adherence to regulatory standards and maintain operational integrity. These audits serve to identify and rectify any potential deviations from established protocols, thereby bolstering the quality assurance framework governing 3DP of paediatric medications.

Drug product validation according to the Ph. Eur. and GMPs, is imperative to ascertain compliance of the preparation with established pharmaceutical principles and standards. While the regulatory guidelines for the incorporation of 3DP technologies within clinical trial settings remain ambiguous, adherence to existing regulatory standards in pharmaceutical manufacturing remains imperative [86, 87, 113]. Regulatory compliance encompasses a multifaceted approach essential for the seamless integration of 3DP of paediatric medicines into the (hospital) pharmaceutical facility. This entails meticulous adherence to GMP guidelines, rigorous compliance with pertinent pharmaceutical regulations and standards, and the acquisition of requisite approvals from regulatory authorities such as the AEMPS [44, 85, 113]. These approvals validate the adherence to regulatory standards and signify authorization for the manufacturing and administration of medications produced via 3DP technologies in the clinical trial context. Therefore, adhering to present regulations and working towards new or future regulations specifically for point-of-care 3DP technologies of medicines is essential [47].

## Learning experiences from the authors and associated regulatory body

**Fig. 4:**
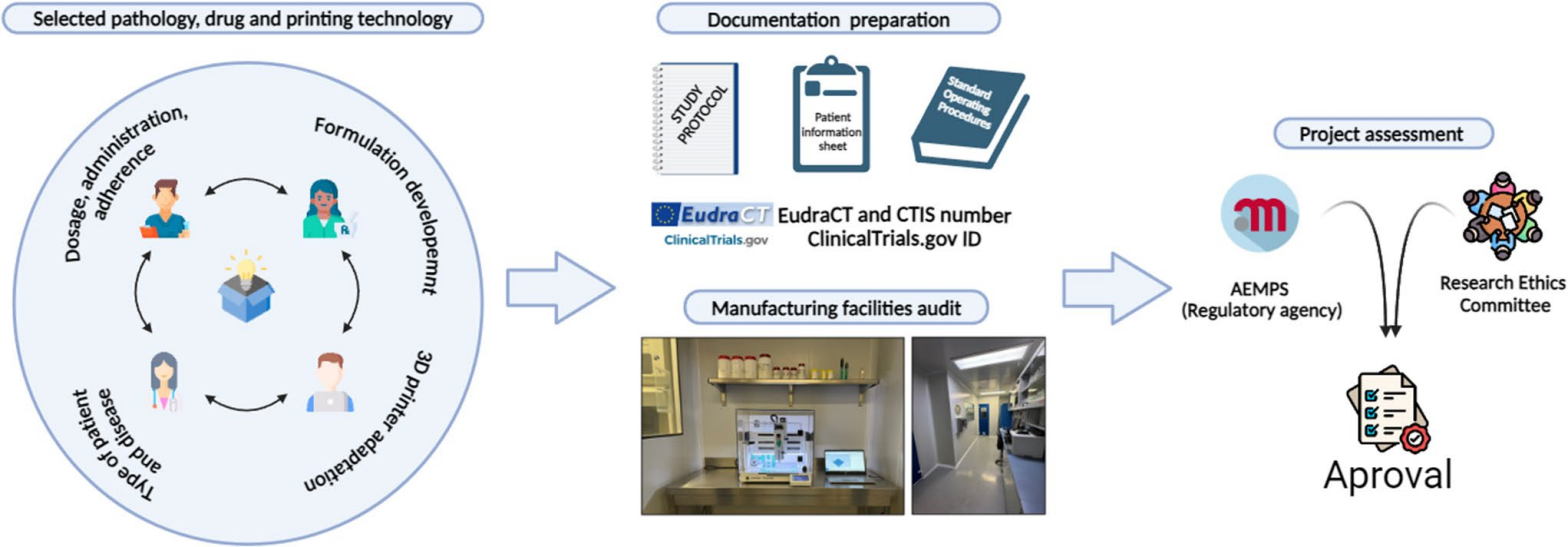
Graphical representation of the entire study development process. AEMPS: Spanish Agency for Medicine and Health Products. CTIS: Clinical Trials Information System

A summarized graphical representation of the entire process explained below can be found in Fig. 4. In 2021, the conceptualization of the study in question began, involving the selection of the pathology, the drug, and the printing method. This stage involved a multidisciplinary team including pharmacists, doctors, hardware and software engineers, and formulation scientists. The pharmacists’ perspective identified the need for a new formulation to improve tolerability and adherence for the specific disease. Together with the medical team, both contributed their expertise to confirm the disease, patient type and age, and dosage. Hardware and software engineers adapted the 3D printer and its controlling software to meet GMP requirements and the needs of the end-user. Formulation scientists developed the formulation and provided their opinion on the most suitable 3D printing technology for the selected drug in the clinical trial. As previously explained, the selected pathology for the clinical trial development was adrenal insufficiency, and the chosen drug was hydrocortisone as it meets almost all requirements explained. SSE printing technology was selected because this technique does not require high temperatures that could degrade hydrocortisone. It is a cleaner process compared to other technologies and allows for the development of printlets that can be chewed, in case of need, by paediatric patients. More information related to the development of hydrocortisone pharma-ink and the printing method can be found in a previous article published by this team [81].

Subsequent stages involved the development of the study protocol and the preparation of essential documentation (for example patient information sheet and SOPs). Considering that the study is a clinical trial with drug manufacturing in compliance with current regulations in Spain [44, 113], an audit of the manufacturing room of the hospital pharmacy department was necessary. An audit request was, therefore, submitted to the Directorate General of Health Planning and Regulation of Catalonia. Owing to the constraints imposed by the COVID-19 pandemic, the audit was conducted remotely through a review of documents. Following the receipt of audit authorization and the completion of all preparatory steps, the study sought and obtained approval from the Research Ethics Committee of Vall d’Hebron Barcelona Hospital and the AEMPs. By early 2022, with favorable assessments, the trial received authorization (EudraCT number: 2021-001069-20) to proceed. Subsequent to this authorization, the initiation of the clinical trial was widely media publicized at the outset of 2023 [117–119]. This significant media exposure prompted the AEMPs to request a halt of the clinical trial commencement to reassess the documentation to be sure the 3DP manufacturing method would not have a negative impact in the participants of the study.

From that moment onward, throughout the first half of 2023, a series of meetings was held with AEMPs experts from various departments (Division of Chemistry and Pharmaceutical Technology, Clinical Trials Area, Division of Pharmacology and Clinical Evaluation and Department of Inspection and Control) to further scrutinize the prerequisites for initiating the study with this innovative manufacturing technique (3DP). Additional evaluations and tests of the pharma-ink were required, accompanied by another facility audit. Following numerous discussions and obtaining additional data from formulation assessments, including new pH dilution tests and other analyses, the authors presented a Substantial Modification of the clinical trial. The AEMPs endorsed the findings, thus allowing the trial to commence after the approval of the new facility audit. In early 2024, two auditors from the Directorate General of Health Planning and Regulation of Catalonia conducted a follow-up audit in person at the manufacturing room of the pharmacy department of the hospital. This examination identified minor deviations from GMP standards, including inadequate insect protection, as well as issues with the organization of facilities and documentation. These issues were rectified, and subsequent approval of the audit confirmed the GMP compliance of the hospital’s manufacturing facility. In the meantime, the results from the hydrocortisone pharma-ink and printlets pharmaceutical quality validation were published [81].

**Fig. 5:**
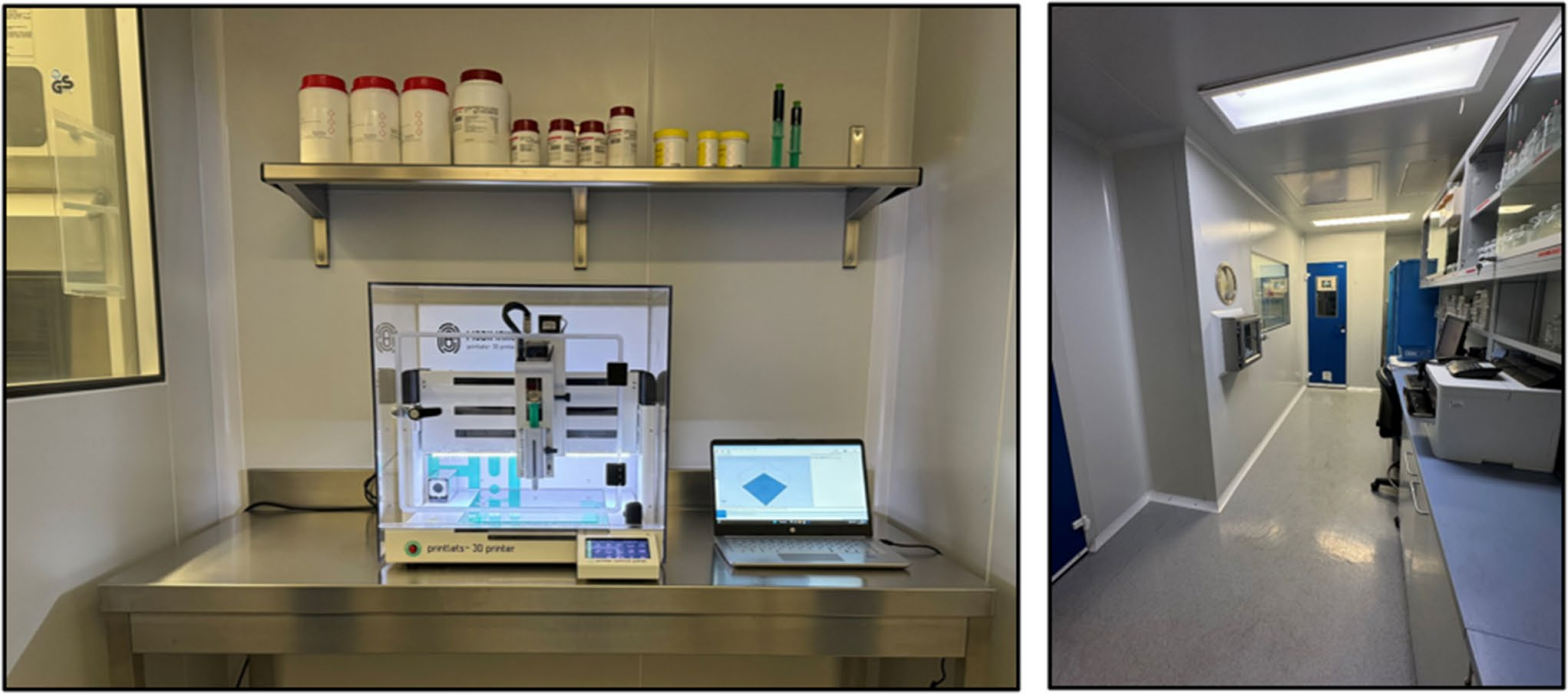
The left image depicts the pharmaceutical 3D printer M3DIMAKER™ in the Good Manufacturing Practice manufacturing room within the hospital pharmacy. The right image shows another section of the manufacturing room, featuring the entrance door to the printer room

Within the hospital, the printer (M3DIMAKER 1, FABRX) was positioned in the manufacturing facility (Fig. 5) of the pharmacy department, conforming to the stringent regulations governing drug manufacturing in clinical trial contexts [44, 113]. At the time of drafting this article, the final endorsement from the AEMPs was obtained (EudraCT number: 2021-001069-20, EUCT Number: 2024-519149-31-00 and ClinicalTrials.gov ID: NCT06435481 [54]), to initiate patient recruitment in early 2025.

## Conclusions

This article evaluated the main factors affecting the use of 3DP technology to manufacture personalized medicines within a hospital setting in a clinical trial. Additionally, it provides a comprehensive guide for obtaining clinical trial approval from the AEMPS to produce personalized 3D printed medicines at the point-of-care in Spain.

The manuscript highlights the importance of selecting a suitable disease and drug/s for clinical trial development; patient recruitment characteristics and requirements; equipment, facilities and printing technology for compounding; and regulatory requirements for the clinical trial. All performed tests and obtained results regarding the pharmaceutical development of the pharma-ink and printing process validation need to be included in the investigational medicinal product dossier (IMPD) and submitted to the AEMPS to demonstrate the quality of the manufactured medicines to be administered to patients in the clinical trial.

This work aims to contribute to international harmonization efforts, serving as a reference for the implementation of similar studies in other countries.

## Data Availability

The datasets generated during and/or analysed during the current study are available from the corresponding author on reasonable request.
